# Learning modular policies for robotics

**DOI:** 10.3389/fncom.2014.00062

**Published:** 2014-06-11

**Authors:** Gerhard Neumann, Christian Daniel, Alexandros Paraschos, Andras Kupcsik, Jan Peters

**Affiliations:** ^1^Department of Computer Science, Intelligent Autonomous Systems, Technische Universität DarmstadtDarmstadt, Germany; ^2^School of Computing, National University of SingaporeSingapore; ^3^Empirical Inference, Intelligent Systems, Max Planck InstituteTübingen, Germany

**Keywords:** robotics, policy search, modularity, movement primitives, motor control, hierarchical reinforcement learning

## Abstract

A promising idea for scaling robot learning to more complex tasks is to use elemental behaviors as building blocks to compose more complex behavior. Ideally, such building blocks are used in combination with a learning algorithm that is able to learn to select, adapt, sequence and co-activate the building blocks. While there has been a lot of work on approaches that support one of these requirements, no learning algorithm exists that unifies all these properties in one framework. In this paper we present our work on a unified approach for learning such a modular control architecture. We introduce new policy search algorithms that are based on information-theoretic principles and are able to learn to select, adapt and sequence the building blocks. Furthermore, we developed a new representation for the individual building block that supports co-activation and principled ways for adapting the movement. Finally, we summarize our experiments for learning modular control architectures in simulation and with real robots.

## 1. Introduction

Robot learning approaches such as policy search methods (Kober and Peters, 2010; Kormushev et al., 2010; Theodorou et al., 2010) have been very successful. Kormushev et al. (2010) Learned to flip pan-cakes and Kober and Peters (2010) Learned the game ball-in-the-cup. Despite these impressive applications, robot learning still offers many challenges due to the inherent high-dimensional continuous state and action spaces, the high costs of generating new data with the real robot, the partial observability of the environment and the risk of damaging the robot due to overly aggressive exploration strategies. These challenges have, so far, prevented robot learning methods to scale to more complex real world tasks.

However, many motor tasks are heavily structured. Exploiting such structures may well be the key to scale robot learning to more complex real world domains. One of the most common structures of a motor task is modularity. Many motor tasks can be decomposed into elemental movements or movement primitives (Schaal et al., 2003; Khansari-Zadeh and Billard, 2011; Rozo et al., 2013) that are used as building blocks in a modular control architecture. For example, playing tennis can be decomposed into single stroke-based movements, such as a forehand and a backhand stroke. To this end, we need a learning architecture that learns to select, improve, adapt, sequence and co-activate the elemental building blocks. Adaptation is needed as such building blocks are only useful if they can be reused for a wide range of situations, and, hence the building block needs to be adapted to the current situation. For example, for playing tennis, the ball will always approach the player slightly differently. Furthermore, we need to learn how to sequence such parametrized building blocks. Taking up our tennis example, we need to execute a sequence of strokes such that the opponent player can not return the ball on the long run. For sequencing the building blocks, we ideally want to be able to continuously switch from one building block to the next to avoid abrupt transitions, also called “blending” of building blocks. Finally, co-activation of the building blocks would considerably increase the expressibility of the control architecture. Coming back to the tennis example, co-activating primitives that are responsible for the upper body movement, i.e., the stroke, and primitives that are responsible for the movement of the lower body, i.e., making a side step or a forward step would significantly reduce the number of required building blocks.

In this paper we present an overview over our work that concentrates on learning such modular control architectures by reinforcement learning. We developed new policy search methods that can select and adapt the individual building blocks to the current situation, learn and improve a large number of different building blocks as well as to learn how to sequence building blocks to solve a complex task. Our learning architecture is based on an information-theoretic policy search algorithm called Relative Entropy Policy Search (REPS) proposed by Peters et al. (2010). The main insight used by REPS is that the relative entropy between the trajectory distributions of two subsequent policies during policy search should be bounded. This bound is particularly useful in robotics as it can cope with many of the mentioned challenges of robot learning. It decreases the danger of damaging the robot as the policy updates stay close to the “data” generated by the old policy and do not perform wild exploration. Moreover, it results in a smooth learning process and prevents the algorithm from getting stuck prematurely in local minima even for high dimensional parameter spaces that are typically used in robotics (Peters and Schaal, 2008; Daniel et al., 2012a). While there are several other policy search approaches which can either learn the selection (da Silva et al., 2012), adaptation (Kober et al., 2010b; Ude et al., 2010) or the sequencing (Stulp and Schaal, 2011) of individual building blocks, to the best of our knowledge, our approach offers the first framework that unifies all these properties in a principled way.

**Figure 1:**
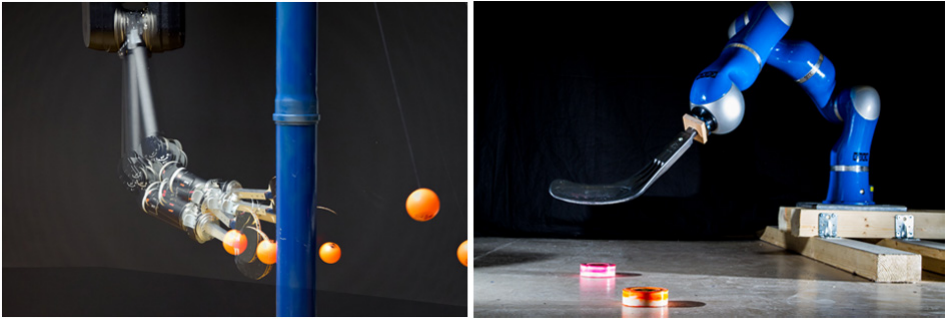
**(Left)** The Barret WAM playing the game of tetherball. **(Right)** The KUKA lightweight arm playing a modified a version of hockey.

A common way to implement the building blocks is to use movement primitives (MPs). Movement primitives provide a compact representation of elemental movements by either parameterizing the trajectory (Schaal et al., 2003; Neumann, 2011; Rozo et al., 2013), muscle activation profiles (dAvella and Pai, 2010) or directly the control policy (Khansari-Zadeh and Billard, 2011). All of these representations offer several advantages, such as the ability to learn the MP from demonstration (Schaal et al., 2003; Rozo et al., 2013), global stability properties (Schaal et al., 2003), co-activation of multiple primitives (dAvella and Pai, 2010), or adaptability of the representation per hyper-parameter tuning (Schaal et al., 2003; Rozo et al., 2013). However, none of these approaches unifies all the desirable properties of a MP in one framework. We therefore introduced a new MP representation that is particularly well suited to be used in a modular control architecture. Our MP representation is based on distributions over trajectories and is called Probabilistic Movement Primitive (ProMP). It can, therefore, represent the variance profile of the resulting trajectories, which allows us to encode the importance of time points as well as represent optimal behavior in stochastic systems (Todorov and Jordan, 2002). However, the most important benefit of a probabilistic representation is that we can perform probabilistic operators on trajectory distributions, i.e., conditioning for adaptation of the MP and a product of distributions for co-activation and blending of MPs. Yet, such a probabilistic representation is of little use if we cannot use it to control the robot. Therefore, we showed that a stochastic time-varying feedback controller can be obtained analytically, enabling us to use the probabilistic movement primitive approach as a promising future representation of a building block in modular control architectures. We will present experiments on several real robot tasks such as playing tether-ball and shooting a hockey puck. The robots used for the experiments are illustrated in Figure 1.

### 1.1. Related work

#### 1.1.1. Movement representations

Different elemental movement representations have been proposed in the literature. The most prominent one is the dynamic movement primitive (DMP) approach (Ijspeert and Schaal, 2003; Schaal et al., 2003). DMPs encode a movement in a parametrized dynamical system. The dynamical system is implemented as a second order spring damper system which is perturbed by a non-linear forcing function *f*. The forcing function depends non-linearly on the phase variable *z*_*t*_ which denotes a clock for the movement. The evolution of the phase variable can be made faster or slower by the temporal scaling factor **τ**, which finally also changes the execution speed of the movement. The forcing function is linearly parametrized by a parameter vector **w** and can be easily learned from demonstrations. In addition to the high dimensional parameters **w**, we can adjust meta-parameters of the DMPs such as the goal attractor **g** of the spring-damper system and temporal scaling factor. In Kober et al. (2010a), the DMPs have been extended to include the final desired velocity in its meta-parameters. DMPs have several advantages. They are easy to learn from demonstrations and by reinforcement learning, they can be used for rhythmic and stroke-based movements and they have build-in stability guarantees. However, they also suffer from some disadvantages. The can not represent optimal behavior in a stochastic environment. In addition, the generalization to a new end position is based on heuristics and not learned from demonstrations and it is not clear how DMPs can be combined simultaneously. Several other movement primitive representation have been proposed in the literature. Some of them are based on DMPs to overcome their limitations (Calinon et al., 2007; Rozo et al., 2013), but none of them can overcome all the limitations in one framework. Rozo et al. (2013) estimate a time varying feedback controller for the DMPs, however, how this feedback controller is obtained is based on heuristics. They also implement a combination of primitives as a product of GMMs which is similar to the work presented here on the probabilistic movement primitives. However, this approach is lacking a principled way of determining a feedback controller that exactly matches the trajectory distribution. Therefore, it is not clear what the result of this product is if we apply the resulting controller on the robot.

Most of the movement representations explicitly depend on time (Ijspeert and Schaal, 2003; Neumann and Peters, 2009; Paraschos et al., 2013; Rozo et al., 2013). For time-dependent representations, a linear controller is often sufficient to model complex behavior as the non-linearity is induced by the time dependency. In contrast, time-independent models such as the Stable Estimator of Dynamical Systems (SEDS) approach (Khansari-Zadeh and Billard, 2011) directly estimate a state dependent policy that is independent of time. Such models require more complex, non-linear controllers. For example, the SEDS approach uses a GMM to model the policy. The GMM is estimated such that the resulting policy is proofed to be stable. Due to the simplicity of the policy, time-dependent representations can be easily scaled up to higher dimensions as shown by Ijspeert and Schaal (2003). Due to the increased complexity, time-independent models are typically used for lower dimensional movements such as modeling the movement directly in task space. Yet, a time-independent model is the more general representation as it does not require the knowledge of the current time step. In this paper, we will nevertheless concentrate on time-dependent movement representations.

#### 1.1.2. Policy search

The most common reinforcement learning approach to learn the parameters of an elemental movement representation such as a DMP is policy search (Williams, 1992; Peters and Schaal, 2008; Kober and Peters, 2010; Kober et al., 2010a). The goal of policy search is to find a parameter vector of the policy such that the resulting policy optimizes the expected long-term reward. Many policy search methods use a stochastic policy for exploration. They can be coarsely categorized according their policy update strategy. Policy gradient methods (Williams, 1992; Peters et al., 2003) are one of the earliest policy update strategies that were applied to motor primitive representations. They estimate the gradient of the expected long-term reward with respect to the policy parameters (Williams, 1992) and update the policy parameters in the direction of this gradient. The main disadvantages of policy gradient methods are the necessity to specify a hand-tuned learning rate, the poor learning speed and that typically many samples are required to obtain a new policy without sample re-use.

More recent approaches rely on probabilistic methods. These methods typically base their derivation on the expectation-maximization algorithm (Vlassis et al., 2009; Kober and Peters, 2010) and formulate the policy search problem as inference problem by transforming the reward into an improper probability distribution, i.e., the transformed reward is required to be always positive. Such transformation is typically achieved by an exponential transformation with a hand-tuned temperature. The resulting policy update can be formulated as a weighted model fitting task where each sample is weighted by the transformed long-term rewards (Kober and Peters, 2010). Using a probabilistic model fitting approach to compute the policy update results in the important advantage that we can use a big toolbox of algorithms for estimating structured probabilistic models, such as the expectation maximization algorithm (Dempster et al., 1977) or variational inference (Neal and Hinton, 1998). Additionally, it does not require a user specified learning rate. These approaches typically directly explore in the parameter space of the policy by estimating a distribution over the policy parameters. Such approach works well if we have a moderate number of parameters.

Another algorithm that has recently gained a lot of attention is the policy improvement by path integrals (PI^2^) algorithm (Theodorou et al., 2010; Stulp and Sigaud, 2012). The path integral theory allows to compute the globally optimal trajectory distribution along with the optimal controls without requiring a value function as opposed to traditional dynamic programming approaches. However, the current algorithm is limited to learning open-loop policies (Theodorou et al., 2010; Stulp and Sigaud, 2012) and may not be able to adapt the the variance of the exploration policy (Theodorou et al., 2010).

#### 1.1.3. Generalization of skills

An important requirement in a modular control architecture is that we can adapt a building block to the current situation or task. We will describe a task or a situation with a context vector **s**. The context vector can contain the objectives of the agent, e.g., throwing a ball to a desired target location, or physical properties of the environment. e.g., the mass of the ball to throw. Ude et al. (2010) use supervised learning to generalize movement primitives from a set of demonstrations. Such approach is well suited to generalize a set of demonstrations to new situations, but can not be used to improve the skills upon the demonstration. To alleviate this limitation, da Silva et al. (2012) combines low-dimensional sub-space extraction for generalization and policy search methods for policy improvement. Finding such low-dimensional sub-spaces is an interesting idea that can considerably improve the generalization of the skills. Yet, there is one important limitation of the approach presented in da Silva et al. (2012). The algorithms for policy improvement and skill generalization work almost independently from from each other. The only way they interact is that the generalization is used as initialization for the policy search algorithm when a new task needs to be learned. As a consequence, the method needs to create many roll-outs for the same task/context in order to improve the skill for this context. Such limitation is relaxed by contextual policy search methods (Kober et al., 2010b; Neumann, 2011). Contextual policy search methods explicitly learn a policy that choses the control parameters ***θ*** in accordance to the context vector **s**. Therefore, a different context can be used for each roll-out. Kober et al. (2010b) us a Gaussian Process (GP) for generalization. While GPs have good generalization properties, they are of limited use for policy search as they typically learn an uncorrelated exploration policy. The approach in Neumann (2011) can use a directed exploration strategy, but it suffers from high computational demands.

#### 1.1.4. Sequencing of skills

Another requirement is to learn to sequence the building blocks. Standard policy search methods typically choose a single parameter vector per episode. Hence, such methods can be used to learn the parameters of a single building block. In order to sequence building blocks, we have to learn how to choose multiple parameter vectors per episode. The first approach (Neumann and Peters, 2009) for learning to sequence primitives was based on value-function approximation techniques, which restricted its application on a rather small set of parameters for each primitive. Recently, (Stulp and Schaal, 2011) adapted the path integral approach to policy search to sequence movement primitives. Other approaches (Morimoto and Doya, 2001; Ghavamzadeh and Mahadevan, 2003) use hand-specified sub-tasks to learn the sequencing of elemental skills. Such an approach is limited in its flexibility of the resulting policy and the sub-tasks are typically not easy to define manually.

#### 1.1.5. Segmentation and modular imitation learning

Segmentation (Kulic et al., 2009; Álvarez et al., 2010; Meier et al., 2011) and modular imitation learning (Niekum et al., 2012) is a very important and challenging problem to autonomously extract the structure of the modular control policy from demonstrations. In Meier et al. (2011) and Álvarez et al. (2010), the segmentation is done due to parameter changes in the dynamical system that is supposed to have created the motion. In Chiappa and Peters (2010), Bayesian methods are used to construct a library of building blocks. Repeated skills are modeled to be generated by one of the building-blocks, which are rescaled and noisy. Based on the segmentation of the demonstrations, we can infer the single building blocks from the data by clustering the segments. One approach that integrates clustering and segmentation is to use Hidden Markov Models (HMMs). Williams and Storkey (2007) used a HMM to extract movement primitives from hand-writing data. While this is a very general approach, it has only been used to rather low-dimensional data, i.e., 2-D movements. Niekum et al. (2012) use a beta-process auto regressive HMM to estimate the segmentation which has the advantage the number of building blocks can also be inferred from data. DMPs are used to represent the policy of the single segments. Butterfield et al. (2010) use a HMM to directly estimate the policy. For each hidden state, they fit a Gaussian Process model to represent the policy of this hidden state. The advantages of these imitation learning approaches is that we can also estimate the temporal structure of the modular control policy, i.e., when to switch from one building block to the next. So far, such imitation learning approaches have not been integrated in a reinforcement learning framework, which seems to be a very interesting direction. For example, in current reinforcement learning approaches, the duration of the building blocks is specified by a single parameter. Estimating the duration of the building blocks from the given trajectory data seems to be a fruitful and more general approach.

## 2. Information theoretic policy search for learning modular control policies

In this section we will sequentially introduce our information theoretic policy search framework used for learning modular control policies. We start our discussion with the adaptation of a single building block. Subsequently, we discuss how to learn to select a building block and, finally, we will discuss sequencing of building blocks.

After introducing each component of our framework, we briefly discuss related experiments on real robots and in simulation. In this paper, we can only give a brief overview over the experiments. For more details, we refer to the corresponding papers. In our experiments with our information theoretic policy search framework, we used Dynamic Movement Primitives (DMP) introduced in Schaal et al. (2003) as building blocks in our modular control architecture. In all our experiments, we used the hyper-parameters of a DMP as parameters of the building blocks, such as the final positions and velocities of the joints (Kober et al., 2010a) as well as the temporal scaling factor of the DMPs for changing the execution speed of the movement.

### 2.1. Learning to adapt the individual building blocks

We formulate the learning of the adaptation of the building blocks as contextual policy search problem (Kober et al., 2010b; Neumann, 2011; Daniel et al., 2012a), where we will for now assume that we want to execute only a single building block. Adaptation of a building block is implemented by an upper-level policy π(***θ*** | **s**) that chooses the parameter vector ***θ*** of the building block according to the current context vector **s**. The context describes the task. It might contain objectives of the agent or properties of the environment, for example, the incoming velocity of a tennis ball. After choosing the parameters ***θ***, the lower level policy **u**_*t*_ = *f*(**x**_*t*_, ***θ***) of the building block takes over and is used to control the robot. Note that we use the symbol **x**_*t*_ to denote the state of the robot. The state **x**_*t*_ typically contains the joint angles **q**_*t*_ and joint velocities $\dot{q}$_*t*_ of the robot and it should not be confused with the context vector **s**. The context vector **s** describes the task and contains higher level objectives of the agent. For example, such a lower level policy can be defined by a trajectory tracking controller that tracks the desired trajectory of a dynamic movement primitive (DMP) (Schaal et al., 2003).

Our aim is to learn an upper-level policy that maximizes the expected reward

(1) $$\begin{array}{l} \;J_{\pi }=\int \text{​ ​}\int \mu (s)\pi (\theta |s)R(s,\theta )dsd\theta ,\\ R(s,\theta )=\int p(\tau |s,\theta )r(\tau ,s)d\tau , \end{array}$$

where *R*(**s**, ***θ***) is the expected reward of the resulting trajectory **τ** when using parameters ***θ*** in context **s** and μ(**s**) denotes the distribution over the contexts that is specified by the learning problem. The distribution *p*(**τ** | **s**, ***θ***) denotes the probability of a trajectory given **s** and ***θ*** and *r*(**τ**, **s**) a user-specified reward function that depends on the trajectory **τ** and on the context **s**. We use the Relative Entropy Policy Search (REPS) algorithm (Peters et al., 2010) as underlying policy search method, The basic idea of REPS is to bound the relative entropy between the old and the new parameter distribution. Here, we will consider the episode-based contextual formulation of REPS (Daniel et al., 2012a; Kupcsik et al., 2013) that is tailored for learning such an upper-level policy. The policy update step is defined as constrained optimization problem where we want to find the distribution *p*(**s**, ***θ***) = μ(**s**) π(***θ*** | **s**) that maximizes the average reward given in Eq. (1) with respect to *p*(**s**, ***θ***) and simultaneously satisfies several constraints. We will first discuss these constraints and show how to compute *p*(**s**, ***θ***). Subsequently, we will explain how to obtain the upper-level policy π(***θ*** | **s**) from *p*(**s**, ***θ***).

Generally, we initialize any policy search (PS) method with an initial policy *q*_0_(**s**, ***θ***) = μ(**s**) *q*_0_(***θ*** | **s**), either obtained through learning from demonstration or by manually setting a distribution for the parameters. The variance of the initial distribution *q*_0_(**s**, ***θ***) defines the exploration region. Policy search is an iterative process. Given the sampling distribution *q*_0_(**s**, ***θ***), we obtain a new distribution *p*_1_(**s**, ***θ***). Subsequently, *p*_1_ is used as new sampling policy *q*_1_ and the process is repeated.

PS methods need to find a trade-off between keeping the initial exploration and constricting the policy to a (typically local) optimum. In REPS, this trade-off is realized via the Kullback-Leibler (KL) divergence. REPS maximizes the reward under the constraint that the KL-divergence to the old exploration policy is bounded, i.e.,

(2) ϵ≥KL(p(s,θ)||q(s,θ)).

Due to this bound, we can choose between exploitation with the greedy policy (high KL-bound) or continue to explore with the old exploration policy (very small KL-bound). The KL divergence in REPS bounds not only the conditional probability π(***θ*** | **s**), i.e., the differences in the policies, but also the joint state-action probabilities *p*(**s**, ***θ***) to ensures that the observed state-action region does not change rapidly over iterations, which is paramount to a real robot learning algorithm. Using the (asymmetric) KL divergence KL (*p*(**s**, ***θ***) ‖ *q*(**s**, ***θ***)) allows us to find a closed form solution of the algorithm. Such closed form would not be possible with the opposite KL divergence, i.e., KL (*q*(**s**, ***θ***) ‖ *p*(**s**, ***θ***)).

We also have to consider that the context distribution *p*(**s**) = ⨜ *p*(**s**, ***θ***) *d**θ*** cannot be freely chosen by the agent as it is specified by the learning problem and given by μ(**s**). Hence, we need to add the constraints ∀ **s** : *p*(**s**) = μ(**s**) to match the given context distribution μ(**s**). However, for continuous context vector **s**, we would end up with infinitely many constraints. Therefore, we resort to matching feature averages instead of single probability values, i.e., ⨜ *p*(**s**) ϕ(**s**) *d***s** = $\hat{\phi }$, where **ϕ**(**s**) is a feature vector describing the context and$\hat{\phi }$ is the mean observed feature vector.

The resulting constrained optimization problem is now given by

(3) $$\begin{array}{l} \max_{p}\int_{s}\int_{\theta }p(s,\theta )R(s,\theta )dsd\theta ,\text{ s}.\text{t}.:\;\epsilon \geq \text{KL}\left(p(s,\theta )||q(s,\theta )\right),\\ \;\int_{s}p(s)\phi (s)ds=\hat{\phi }\;\int_{s}\int_{\theta }p(s,\theta )dsd\theta =1. \end{array}$$

It can be solved by the method of Lagrangian multipliers and yields a closed-form solution solution for *p* that is given by

(4) $$p(s,\theta )\propto q(s,\theta )\exp\left(\frac{R(s,\theta )-V(s)}{\eta }\right),$$

where *V*(**s**) = **v**^*T*^ ϕ(**s**) is a context dependent baseline that is subtracted from the the reward signal. The scalar η and the vector **v** are Lagrangian multipliers that can be found by optimizing the dual function *g*(η, **v**) (Daniel et al., 2012a). It can be shown that *V*(**s**) can be interpreted as value function (Peters et al., 2010) and, hence, estimates the mean performance of the new policy in context **s**.

The optimization defined by the REPS algorithm is only performed on a discrete set of samples *D* = {**s**^[*i*]^, ***θ***^[*i*]^, *R*^[*i*]^}, *i* = 1, …, *N*, where *R*^[*i*]^ denotes the return obtained by the *i*th rollout. The resulting probabilities *p* (**s**^[*i*]^, ***θ***^[*i*]^), see Equation (4), of these samples are used to weight the samples. In order to obtain the weight *p*^[*i*]^ for each sample, we need to divide *p* (**s**^[*i*]^, ***θ***^[*i*]^) by the sampling distribution *q*(**s**, ***θ***) to account for the sampling probability (Kupcsik et al., 2013), i.e.,

(5) $$p^{\left[i\right]}=\frac{p\left(s^{\left[i\right]},\theta^{\left[i\right]}\right)}{q(s^{\left[i\right]},\theta^{\left[i\right]})}\propto \exp\left(\frac{R(s,\theta )-V(s)}{\eta }\right).$$

Hence, being able to sample from *q* is sufficient and *q* is not needed in its analytical form.

**Figure 2:**
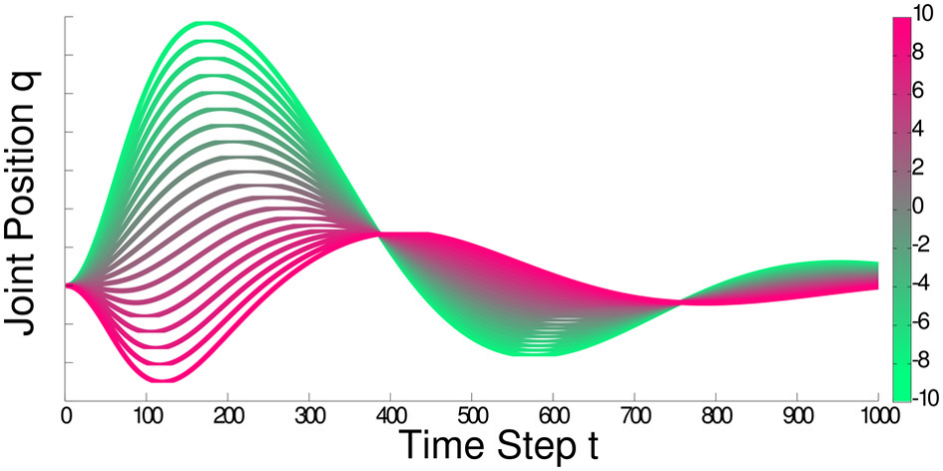
**The figure illustrates the joint trajectories that can be generated when using a linear Gaussian to adapt the DMP parameters according to a one dimensional context variable**. In this illustration, we show the color coding for the context variable in the color bar on the right and show how the generated trajectories change in the main plot. For this plot, we assumed no exploration noise and adapted ten basis functions of the DMP. As we can see, complex behavior can emerge already with a linear adaptation model due to the high-dimensionality of the parameter space.

The upper-level policy π(***θ*** | **s**) is subsequently obtained by performing a weighted maximum-likelihood (ML) estimate. We use a linear-Gaussian model to represent the upper-level policy π(***θ*** | **s**) = 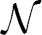 (***θ*** | **a** + **As**, **Σ**) of the building block, where the parameters **a**, **A** and **Σ** are obtained through the ML estimation. As a building block is typically reused only for similar contexts **s**, a linear model is sufficient in most cases. Figure 2 shows an illustration of how a linear model can adapt the trajectories generated by a DMP. In practice, we still need an initial policy *q*. This initial policy can either be obtained through learning from demonstration or by selecting reasonable parameters and variance if the experimenter has sufficient task knowledge.

In Kupcsik et al. (2013), we further improved the data-efficiency of our contextual policy search algorithm by learning probabilistic forward models of the real robot and its environment. With these forward models, we can predict the reward *R* (**s**^[*j*]^, ***θ***^[*j*]^) for unseen context-parameter pairs **s**^[*j*]^ and ***θ***^[*j*]^ and use these additional samples for computing the policy update. The data-efficiency of our method could be improved up to two orders of magnitude using the learned forward models. As we used Gaussian Processes (GPs) (Rasmussen and Williams, 2006) to represent the forward models, this extension of our method is called GPREPS. These forward models were used to generate additional data points that are used for the policy update. For each of these virtual data points, we generated 15 trajectories with the learned forward models. We used the average reward of these predicted trajectories as reward used in the REPS optimization. We used sparse GPs (Snelson and Ghahramani, 2006) to deal with the high number of data points within a reasonable computation time.

#### 2.1.1. Experimental evaluation of the adaptation of building blocks - robot hockey target shooting

**Figure 3:**
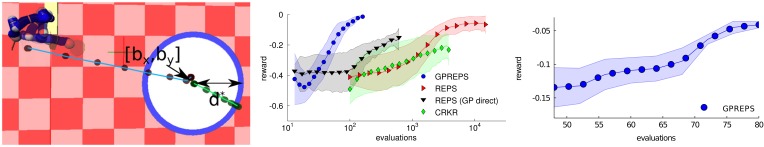
**(Left)** Robot hockey target shooting task. The robot has to shoot a puck at the target puck such that the target puck moves for a specified distance. Both, the initial location of the target puck [*b*_*x*_, *b*_*y*_]^*T*^ and the desired distance d^*^ to move the puck were varied. **(Middle)** Learning curves on the robot hockey task in simulation. GPREPS was able to learn the task within 120 interactions with the environment, while the model-free version of REPS needed about 10000 episodes. **(Right)** GPREPS learning curve on the real robot arm.

In this task we used GPREPS with learned forward models to learn how to adapt the building blocks such that the robot can shoot hockey pucks to different locations. The objective was to make a target puck move for a specified distance by shooting a second hockey puck at the target puck. The context **s** was composed of the initial location [*b*_*x*_, *b*_*y*_]^*T*^ of the target puck and the distance d^*^ that the target puck had to be shoot, i.e., **s** = [*b*_*x*_, *b*_*y*_, d^*^]^*T*^. We chose the initial position of the target puck to be uniformly distributed from the robot's base with displacements *b*_*x*_ ∈ [1.5, 2.5]m and *b*_*y*_ ∈ [0.5, 1]m. The desired displacement context parameter d^*^ is also uniformly distributed d^*^ ∈ [0, 1] m. The reward function

$$r(\tau ,s)=-\underset{t}{\min}||x_{t}-b|{|}_{2}-||d_{T}-d^{*}|{|}_{2}$$

consist of two terms with equal weighting. The first term penalizes missing the target puck located at position **b** = [*b*_*x*_, *b*_*y*_]^*T*^, where the control puck trajectory is ***x***_1:*T*_. The second term penalizes the error in the desired displacement of the target puck, where *d*_*T*_ is the resulting displacement of the target puck after the shot. The parameters ***θ*** define the weights and goal position of the DMP. The policy in this experiment was a linear Gaussian policy. The simulated robot task is depicted in Figure 3.

GPREPS first learned a forward model to predict the initial position and velocity of the first puck after contact with the racket and a travel distance of 20 cm. Subsequently, GPREPS learned the free dynamics model of both pucks and the contact model of the pucks. We assumed that we know the geometry of the pucks to detect a contact. If there is a contact, we used the contact model to predict the state of both pucks after the contact given the state of both pucks before the contact. From this state, we again predicted the final puck positions after they came to stop with a separate GP model.

We compared GPREPS in simulation to directly predicting the reward *R*(**s**, ***θ***), model-free REPS and CrKR (Kober et al., 2010b), a state-of-the-art model-free contextual policy search method. The resulting learning curves are shown in Figure 3 (middle). GPREPS learned the task already after 120 interactions with the environment while the model-free version of REPS needed approximately 10000 interactions. Directly predicting the rewards from parameters ***θ*** using a single GP model resulted in faster convergence but the resulting policies still showed a poor performance (*GP direct*). The results show that CrKR could not compete with model-free REPS. The learned movement is shown in Figure 3 for a specific context. After 100 evaluations, GPREPS placed the target puck accurately at the desired distance with an error ≤ 5 cm.

Finally, we evaluated the performance of GPREPS on the hockey task using a real KUKA lightweight arm. The learning curve of this experiment is shown in Figure 3 (right) and confirms that GP-REPS can find high-quality policies within a small amount of interactions with the environment.

### 2.2. Learning to select the building blocks

In order to select between several building blocks ø, we add an additional level of hierarchy on top of the upper-level policies of the individual building blocks. We assume that each building block shares the same parameter space. The parameters are now selected by first choosing the building block to execute with a gating policy π_*G*_(*o* | **s**) and, subsequently, the upper level parameter policy π_*P*_(***θ*** | **s**, *o*) of the building block *o* selects the parameters ***θ***. Hence, π(***θ*** | **s**) can be written as hierarchical policy

(6) $$\pi (\theta |s)=\sum_{o}\pi_{G}(o|s)\pi_{P}(\theta |s,\;o).$$

In this model, the gating policy composes a complex, non-linear parameter selection strategy out of the simpler upper level policies of the building blocks. Moreover, it can learn multiple solutions for the same context, which also increases the versatility of the learned motor skill (Daniel et al., 2012b). While a similar decomposition in gating policy and option policies has been presented in da Silva et al. (2012), their framework was not integrated in a reinforcement learning algorithm, and hence, generalization and improvement the building blocks is performed by two independent algorithms, resulting in sample-inefficient policy updates.

To incorporate multiple building blocks, we now bound the Kullback-Leibler divergence between *q*(**s**, ***θ***, *o*) and *p*(**s**, ***θ***, *o*). As we are interested in versatile solutions, we also want to avoid that several building blocks concentrate on the same solution. Hence, we want to limit the “overlap” between building blocks in the parameter space. In order to do so, we bound the expected entropy of the conditional distribution *p*(*o* | **s**, ***θ***), i.e.,

(7) $$-\int p(s,\theta )\sum_{o}p(o|s,\theta )\log p(o|s,\theta )dsd\theta \leq \kappa .$$

A low entropy of *p*(*o* | **s**, ***θ***) ensures that our building blocks do not overlap in parameter space and, thus, represent individual and clearly separated solutions (Daniel et al., 2012a). The new optimization program results in the hierarchical version of REPS, denoted as HiREPS. We can again determine a closed form solution for *p*(**s**, ***θ***, *o*) which is given in Daniel et al. (2012a). As in the previous section, the optimization problem is only solved for a given set of samples that has been generated from the distribution *q*(**s**, ***θ***). Subsequently, the parameters of the gating policy and the upper-level policies are obtained by weighted ML estimates. We use a Gaussian gating policy and an individual linear Gaussian policy π(***θ*** | **s**, *o*) = 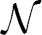 (***θ*** | **a**_*o*_, + **A**_*o*_**s**, **Σ**_*o*_) for each building block. As we use a linear upper-level policy and the used DMPs produce only locally valid controllers, our architecture might require a large number of building blocks.

#### 2.2.1. Experimental evaluation of the selection of building blocks - robot tetherball

**Figure 4:**
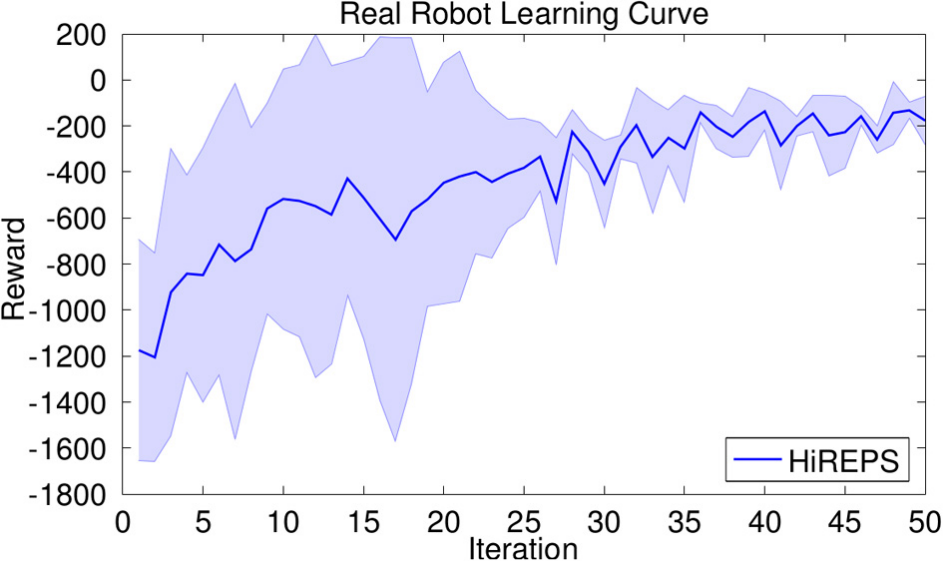
**Average rewards for learning tetherball on the real robot**. Mean and standard deviation of three trials. In all of the three trials, after 50 iterations the robot has found solutions to wind the ball around the pole on either side.

**Figure 5:**
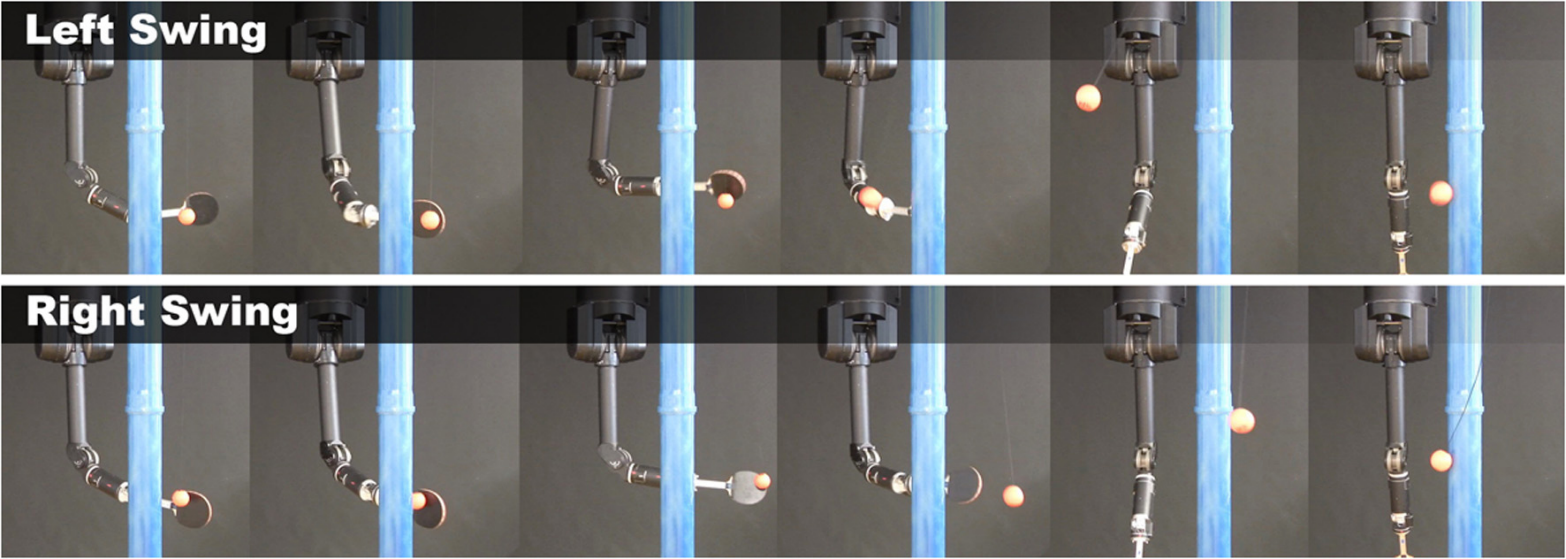
**Time series of a successful swing of the robot**. The robot first has to swing the ball to the pole and, subsequently, when the ball has swung backwards, can arc the ball around the pole. The movement is shown for a shoot to the left and to the right of the pole.

In robot tetherball, the robot has to shoot a ball that is fixed with a string on the ceiling such that it winds around a pole. The robot obtains a reward proportional to the speed of the ball winding around the pole. There are two different solutions, to wind the ball around the left or to the right side of the pole. Two successful hitting movements of the real robot are shown in Figure 5. We decompose our movement into a swing-in motion and a hitting motion. As we used the non-sequential algorithm for this experiment, we represented the two motions by a single set of parameters and jointly learn the parameters ***θ*** for the two DMPs. We start the policy search algorithm with 15 options with randomly distributed parameters sampled from a Gaussian distribution around the parameters of the initial demonstration. We use a higher number of building blocks to increase the probability of finding both solutions with the building blocks. If we use two randomly initialized building blocks, the probability that both cover the same solution is quite high. We delete unused building blocks that have a very small probability of being chosen, i.e., *p*(*o*) < 0.001. The learning curve is shown in Figure 4 (left). The noisy reward signal is mostly due to the vision system and partly also due to real world effects such as friction. Two resulting movements of the robot are shown in Figure 5. The robot could learn a versatile strategy that contained building blocks that wind the ball around the left and building blocks that wind the ball around the right side of the pole.

### 2.3. Learning to sequence the building blocks

To execute multiple building blocks in a sequence, we reformulate the problem of sequencing building blocks as Markov Decision Process (MDP). Each building block defines a transition probability *p*(**s**' | **s**, ***θ***) over future contexts and an immediate reward function *R*(**s**, ***θ***). It is executed until its termination condition *t*_*o*_(**s**, ***θ***) is satisfied. However, in our experiments, we used a fixed duration for each building block. Note that traditional reinforcement learning methods, such as TD-learning, can not deal with such MDPs as its action space is high dimensional and continuous.

We concentrate on the finite-horizon case, i.e., each episode consists of *K* decision steps where each step is defined as the execution of an individual building block. For clarity, we will only discuss the sequencing of a single building block, however, the selection of multiple building blocks at each decision step can be easily incorporated (Daniel et al., 2013).

In the finite horizon formulation of REPS we want to find the probabilities *p*_*k*_(**s**, ***θ***) = *p*_*k*_(**s**) π(***θ*** | **s**), *k* ≤ *K*, and *p*_*K* + 1_(**s**) that maximize the expected long term reward

$$J=\int_{s}p_{K+1}(s)R_{K+1}(s)ds+\sum_{k=1}^{K}\int_{s}\int_{\theta }p_{k}(s,\theta )R_{k}(s_{k},\theta_{k})dsd\theta ,$$

where *R*_*K* + 1_(**s**_*K* + 1_) denotes the final reward for ending up in the state **s**_*K* + 1_ after executing the last building block. As in the previous case, the initial context distributions is given by the task, i.e., ∀ **s**: *p*_1_(**s**) = μ_1_(**s**). Furthermore, the context distribution at future decision steps *k* > 1 need to be consistent with the the past distributions *p*_*k* − 1_(**s**, ***θ***) and the transition model *p*(**s**' | **s**, ***θ***), i.e.,

$$\forall s^{\prime },\;k>1:p_{k}\left(s^{\prime }\right)=\int_{s}\int_{\theta }p_{k-1}(s,\theta )p\left(s^{\prime }|s,\theta \right)dsd\theta ,$$

for each decision step of the episode. These constraints connect the policies for the individual decision-steps and result in a policy π_*k*_(***θ*** | **s**) that optimizes the long-term reward instead of the immediate ones. As in the previous sections, these constraints are again implemented by matching feature averages.

The closed form solution of the joint distribution *p*_*k*_(**s**, ***θ***) yields

$$\begin{array}{l} p_{k}(s,\theta )\propto q_{k}(s,\theta )\exp\left(\frac{A_{k}(s,\theta )}{\eta_{k}}\right),\\ A_{k}(s,\theta )=R_{k}(s,\theta )+{𝔼}_{p\left(s^{\prime }|s,\theta \right)}\left[V_{k+1}\left(s^{\prime }\right)\right]-V_{k}(s). \end{array}$$

We can see that the reward *R*_*k*_(**s**, ***θ***) is transformed into an advantage function *A*_*k*_(**s**, ***θ***) where the advantage now also depends on the expected value of the next state 𝔼_*p*(**s**' | **s**, ***θ***)_[*V*_*k* + 1_ (**s**')]. This term ensures that we do not just optimize the immediate reward but the long term reward.

#### 2.3.1. Experimental evaluation of sequencing of building blocks - sequential robot hockey

**Figure 6:**
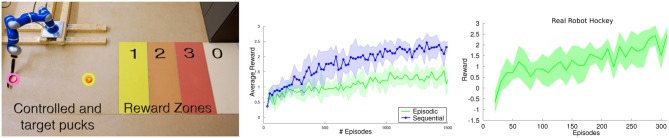
**(Left)** The sequential robot hockey task. The robot has two pucks, the pink control puck and the yellow target puck. The task is to shoot the yellow target puck into one of the colored reward zones. Since the best reward zone is too far away from the robot to be reached with only one shot, each episode consists of three strikes. After each strike, the control puck is returned to the robot, but the target puck is only reset after one episode is concluded. **(Middle)** Comparison of sequential motor primitive learning to the episodic learning setup on the simulated robot hockey task. The sequential motor primitive learning framework was able to find a good strategy to place the puck in the third reward zone in most of the cases while the episodic learning scenario failed to learn such a strategy. **(Right)** One trial of the real robot hockey tasks. The robot starts with a negative initial reward and learns to achieve an average reward of 2.5 after 300 episodes.

We used the sequential robot hockey task to evaluate sequential motor skill learning framework. The robot has to move the target puck into one of three target areas by sequentially shooting a control puck at the target puck. The target areas are defined by a specified distance to the robot, see Figure 6 (left). The robot gets rewards of 1, 2, and 3 for reaching zone 1, 2 or 3, respectively. After each shot, the control puck is returned to the robot. The target puck, however, is only reset after every third shot.

The 2-dimensional position of the target puck defines the context **s** of the task and the parameter vector ***θ*** defines the goal positions of the DMP that define the desired trajectory of the robot's joints. After performing one shot, the agent observes the new context to plan the subsequent shot. In order to give the agent an incentive to shoot at the target puck, we punished the agent with the negative minimum distance of the control puck to the target puck after each shot. While this reward was given after every step, the zone reward was only given at the end of the episode (every third step) as *r*_*K* + 1_(**s**_*K* + 1_).

We compared our sequential motor primitive learning method with its episodic variant on a realistic simulation. For the episodic variant we used one extended parameter vector $\tilde{\theta }$ that contained the parameters for all three hockey shoots. The comparison of both methods can be seen in Figure 6 (middle). Due to the high-dimensional parameter space, the episodic learning setup failed to learn a proper policy while our sequential motor primitive learning framework could learn policies of much higher quality.

On the real robot, we could reproduce the simulation results. The robot learned a strategy which could move the target puck to the highest reward zone in most of the cases after 300 episodes. The learning curve is shown in Figure 6 (right).

## 3. Probabilistic movement primitives

In the second part of this paper, we investigate new representations for the individual building blocks of movements that are particularly suited to be used in a modular control architecture. In all experiments for our modular policy search framework, we so far used the Dynamic Movement Primitive (DMP) approach (Schaal et al., 2003). DMPs are widely used, however, when used for our modular control architecture, DMPs suffer from severe limitations as they do not support co-activation or blending of building blocks. In addition, the DMPs use heuristics for the adaptation of the motion. Hence, we focus our discussion on our new movement primitive (MP) representation (Paraschos et al., 2013) on a these two important properties.

We use a trajectories **τ** = {*q*_*t*_} _*t* = 0… *T*_, defined by the joint angles *q*_*t*_ over time, to model a single movement. We will use a probabilistic representation of a movement, which we call probabilistic movement primitives (ProMP), where a movement primitive describes several ways how to execute a movement (Paraschos et al., 2013). Hence, the movement primitive is given as distribution *p*(**τ**) over trajectories. A probabilistic representation offers several advantages that make it particularly suitable to be used in a modular control architecture. Most importantly, it offers principled ways to adapt as well as to co-activate movement primitives. Yet, these advantages of a probabilistic trajectory representation are of little use if we can not use it to control the robot. Therefore, we derive a stochastic feedback controller in closed form that can exactly reproduce a given trajectory distribution, and, hence, trajectory distributions can be used directly for robot control.

In this section, we present two experiments that we performed with the ProMP approach. As we focused on the representation of the individual building blocks, we evaluated the new representation without the use of reinforcement learning and learned the ProMPs by imitation. In our experiments, we illustrate how to use conditioning as well as co-activation of the building blocks.

### 3.1. Probabilistic trajectory representation

In the imitation learning setup, we assume that we are given several demonstrations in terms of trajectories **τ**_*i*_. In our probabilistic approach we want to learn a distribution of these trajectories. We will first explain the basic representation of a trajectory distribution and subsequently cover the two new operations that are now available in our probabilistic framework, i.e., conditioning and co-activation. Finally, we will explain in Section 3.3 how to control the robot with a stochastic feedback controller that exactly reproduces the given trajectory distribution.

We use a weight vector **w** to compactly represent a single trajectory **τ**. The probability of observing a trajectory ****τ**** given the weight vector **w** is given as a linear basis function model *p*(**τ** | **w**) = ∏_*t*_
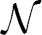
(**y**_*t*_ | **Ψ**^*T*^_*t*_**w**, **Σ**_*y*_), where **y**_*t*_ = [*q*_*t*_, $\dot{q}$_*t*_]^*T*^ contains the joint position *q*_*t*_ and joint velocity $\dot{q}$_*t*_, **Ψ**_*t*_ = [ψ, $\dot{\psi }$_*t*_] defines the time-dependent basis matrix and ϵ_*y*_ is zero-mean i.i.d. Gaussian noise.

We now abstract a distribution over trajectories as distribution *p*(**w**; ***θ***) over the weight vector **w** that is parametrized by the parameter vector ***θ***. The original trajectory distribution *p*(**τ**; ***θ***) can now be computed by marginalizing of the weight vector **w**, i.e., *p*(**τ**; ***θ***) = ⨜ *p*(**τ** | **w**)*p*(**w**; ***θ***)*d***w**. We will assume a Gaussian distribution for *p*(**w**; ***θ***) = 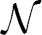
(**w** | μ_*w*_, **Σ**_*w*_) and, hence, *p*(**τ**; ***θ***) can be computed analytically, i.e.,

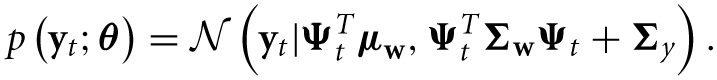



As a probabilistic MP represents multiple ways to execute an elemental movement, we also need multiple demonstrations to learn *p*(**w**; ***θ***). The parameters ***θ*** = {**μ**_**w**_, **Σ**_**w**_} can be learned by maximum likelihood estimation, for example, by using the expectation maximization algorithm (Lazaric and Ghavamzadeh, 2010).

For multi-dimensional systems, we can also learn the coupling between the joints. Coupling is typically represented by the covariance of the joint positions and velocities. We can learn this covariance by maintaining a parameter vector **w**_*i*_ for each dimension *i* and learn a distribution over the combined weight vector **w** = [**w**^*T*^_1_, …, **w**^*T*^_*n*_]^*T*^.

To be able to adapt the execution speed of the movement, we introduce a phase variable *z* to decouple the movement from the time signal (Schaal et al., 2003). The phase can be any function *z*(*t*) monotonically increasing with time. The basis functions ψ_*t*_ are now decoupled from the time and depend on the phase, such that ψ_*t*_ = ψ (*z*_*t*_) and $\dot{\psi }$_*t*_ = ψ'(*z*_*t*_)*ż*_*t*_. The choice of the basis functions depends on whether we want to model rhythmic movements, where we use normalized Von-Mises basis functions that are periodic in the phase, or stroke-based movements, where we use normalized Gaussian basis functions,

(8) $$\begin{array}{l} \phi_{i}^{\text{G}}(z)=\exp\left(-\frac{{\left(z_{t}-c_{i}\right)}^{2}}{2h}\right),\\ \phi_{i}^{\text{VM}}(z)=\exp\left(h\cos(2\pi (z_{t}-c_{i}))\right). \end{array}$$

The parameter *h* defines the width of the basis and *c*_*i*_ the center for the *i*th basis function. We normalize the basis functions **ϕ**_*i*_ with ψ_*i*_(*z*_*t*_) = **ϕ**_*i*_(*z*)/∑_*j*_ **ϕ**_*j*_(*z*).

### 3.2. New probabilistic operators for movement primitives

The probabilistic formulation of MPs enables us to use new probabilistic operators on our movement primitive representation. Adaptation of the movement can be accomplished by conditioning on desired positions or velocities at time step *t*. Co-activation and blending of MPs can be implemented as as product of two trajectory distributions.

#### 3.2.1. Adaptation of the building blocks by conditioning

For efficient adaptation, our building blocks should support the modulation of hyper-parameters of the movements such as the desired final joint positions or the joint positions at given via-points. For example, DMPs allow for the adaptation of the final position by modulation of the point attractor of the system. However, how the final position modulates the trajectory is hard-coded in the DMP-framework and can not be learned from data. This adaptation mechanism might violate other task constraints.

In our probabilistic formulation, such adaptation operations can be described by conditioning the MP to reach a certain state y^*^_t_ at time *t*. Conditioning can be performed by adding a new desired observation **x**_*t*_ = [y^*^_t_, Σ^*^_y_] to our probabilistic model where y^*^_t_ represents the desired position and velocity vector at time *t* and Σ^*^_y_ specifies the accuracy of the desired observation. By applying Bayes theorem, we obtain a new distribution over **w**, i.e., *p*(**w** | x^*^_t_) ∝ 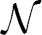 (y^*^_t_ | **Ψ**^*T*^_*t*_**w**, Σ^*^_y_)*p*(**w**). As *p*(**w** | ***θ***) is Gaussian, the conditional distribution *p* (**w** | y^*^_t_) is also Gaussian and can be computed analytically

(9) $$\mu_{w}^{\left[\text{new}\right]}=\mu_{w}+\Sigma_{w}\Psi_{t}{\left(\Sigma_{y}^{*}+\Psi_{t}^{T}\Sigma_{w}\Psi_{t}\right)}^{-1}\left(y_{t}^{*}-\Psi_{t}^{T}\mu_{w}\right),$$

(10) $$\Sigma_{w}^{\left[\text{new}\right]}=\Sigma_{w}-\Sigma_{w}\Psi_{t}{\left(\Sigma_{y}^{*}+\Psi_{t}^{T}\Sigma_{w}\Psi_{t}\right)}^{-1}\Psi_{t}^{T}\Sigma_{w}.$$

**Figure 7:**
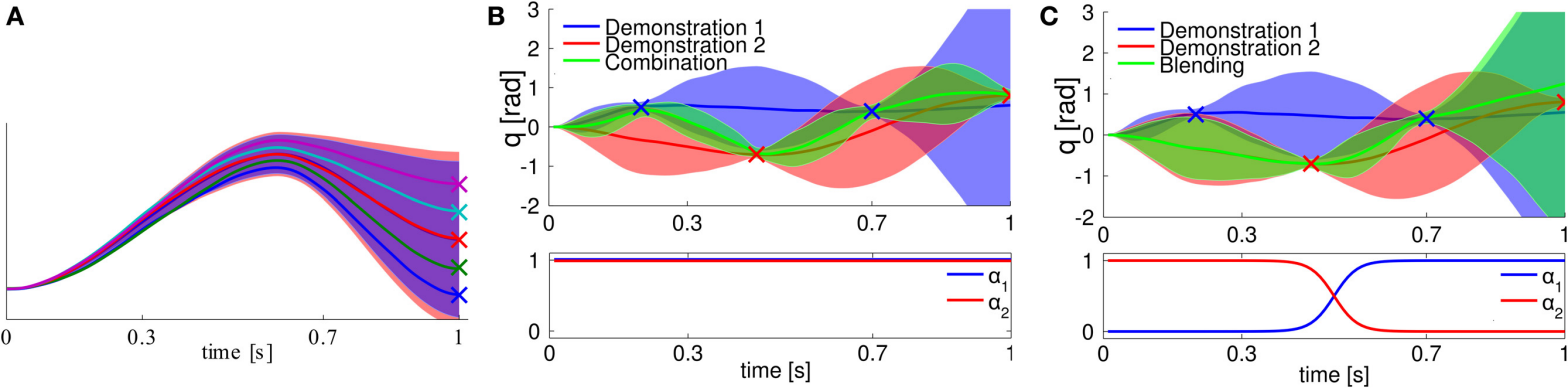
**(A)** Conditioning on different target states. The blue shaded area represents the learned trajectory distribution. We condition on different target positions, indicated by the “x”-markers. The produced trajectories exactly reach the desired targets while keeping the shape of the demonstrations. **(B)** Combination of two ProMPs. The trajectory distributions are indicated by the blue and red shaded areas. Both primitives have to reach via-points at different points in time, indicated by the “x”-markers. We co-activate both primitives with the same activation factor. The trajectory distribution generated by the resulting feedback controller now goes through all four via-points. **(C)** Blending of two ProMPs. We smoothly blend from the red primitive to the blue primitive. The activation factors are shown in the bottom. The resulting movement (green) first follows the red primitive and, subsequently, switches to following the blue primitive.

We illustrated conditioning a ProMP to different target states in Figure 7A. As we can see, the modulation of a target state is also learned from demonstration, i.e., the ProMP will choose a new trajectory distribution that goes through the target state, and, at the same time, is similar to the learned trajectory distribution.

#### 3.2.2. Combination and blending by multiplying distributions

In our probabilistic representation, a single MP represents a whole family of movements. Co-activating two MPs should return a new set of movements which are contained in both MPs. Such operation can be performed by multiplying two distributions. We also want to weight the activation of each primitive *o*_*i*_ by a time-varying activation factor α_*i*_(*t*), for example, to continuously blend the movement execution from one primitive to the next. The activation factors can be implemented by taking the distributions of the individual primitives to the power of α_*i*_(*t*). Hence, the co-activation of ProMPs yields p^*^(**τ**) ∝ ∏_*t*_ ∏ _*i*_*p*_*i*_(**y**_*t*_)^α^_*i*_^(*t*)^.

For Gaussian distributions *p*_*i*_(**y**_*t*_) = 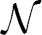
(**y**_*t*_ | μ^[*i*]^_*t*_, **Σ**^[*i*]^_*t*_), the resulting distribution p^*^(**y**_*t*_) is again Gaussian and we can obtain its mean μ^*^_t_ and variance Σ^*^_t_ analytically with variance and mean

(11) $$\begin{array}{l} \Sigma_{t}^{*}={\left(\sum_{{}_{i}}{\left(\Sigma_{t}^{\left[i\right]}/\alpha_{i}(t)\right)}^{-1}\right)}^{-1},\\ \mu_{t}^{*}={\left(\Sigma_{t}^{*}\right)}^{-1}\left(\sum_{{}_{i}}{\left(\Sigma_{t}^{\left[i\right]}/\alpha_{i}(t)\right)}^{-1}\mu_{t}^{\left[i\right]}\right). \end{array}$$

Both terms are required to obtain the stochastic feedback controller that is finally used to control the robot. We illustrated co-activating two ProMPs in Figure 7B and blending of two ProMPs in Figure 7C.

### 3.3. Using trajectory distributions for robot control

In order to use a trajectory distribution *p*(**τ** | ***θ***) for robot control, we have to obtain a controller which can exactly reproduce the given distribution. As we show in Paraschos et al. (2013), such controller can be obtained in closed form if we know the system dynamics $\dot{\text{y}}$ = *f*(**y**, **u**) + ϵ_*y*_ of the robot^1^. We model the controller as time-varying stochastic linear feedback controller, i.e., **u**_*t*_ = **k**_*t*_ + **K**_*t*_ **y**_*t*_ + ϵ_*u*_, where **k**_*t*_ denotes the feed-forward gains, **K**_*t*_ the feedback gains and ϵ_*u*_ ~ 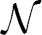
(**0**, **Σ**_*u*_) the controller noise. Hence, the controller is determined by **k**_*t*_, **K**_*t*_ and **Σ**_*u*_ for each time point. All these terms can be obtained analytically by predicting the distribution *p*_model_(**y**_*t* + *dt*_) from *p*(**y**_*t*_ | ***θ***) with the known model of the system dynamics and subsequently matching the moments of *p*(**y**_*t* + *dt*_ | ***θ***) and the moments of the predicted distribution *p*_model_(**y**_*t* + *dt*_). The resulting controller exactly reproduces the given trajectory distribution *p*(**τ** | ***θ***) (Paraschos et al., 2013).

While the ProMP approach has many similarities to the approach introduced in Rozo et al. (2013) by Calinon and colleagues, there are also important differences to this approach. They also learn a trajectory distribution which is modeled with a GMM, where the output variables are the joint angles and the time step *t*. The probability for the joint angles at time step *t* is then obtained by conditioning on *t*. However, it is unclear how to condition on being at a certain state q^*^_t_ at time step, which is very different then just conditioning on being in time step *t*. In this case, the mixture components need to be changed such that the trajectory distribution passes through q^*^_t_ at time step t. How to implement this change with a GMM is an open problem. Note that the ProMP approach is very different from a GMM. It uses a linear basis function model and learns the correlation of the parameters of the basis functions for the different movements. Time is not modeled as random variable but as conditional variable right away. Due to the learned correlations, we can condition on reaching q^*^_t_ at time step *t* and the trajectory distribution smoothly passes through q^*^_t_ with high accuracy.

Furthermore, a trajectory distribution alone is not sufficient to control a robot as it requires a feedback controller that determines the control actions. How to obtain this feedback controller from the trajectory distribution is based on heuristics in Rozo et al. (2013). I.e., when we apply the feedback controller on the real robot, we will not reproduce the learned trajectory distribution. The produced trajectory distribution might be similar, but we do not know how similar. Therefore, for all operations performed on the trajectory distributions (i.e., a combination of distributions by a product), it is hard to quantify the effect of this operation on the resulting motions that are obtained from the heuristic feedback controller. In contrast, the ProMPs come with a feedback controller that exactly matches the trajectory distribution. Hence, for a combination of distributions, we know that the feedback controller will exactly follow the product of the two distributions.

#### 3.3.1. Experimental evaluation of the combination of objectives at different time-points

**Figure 8:**
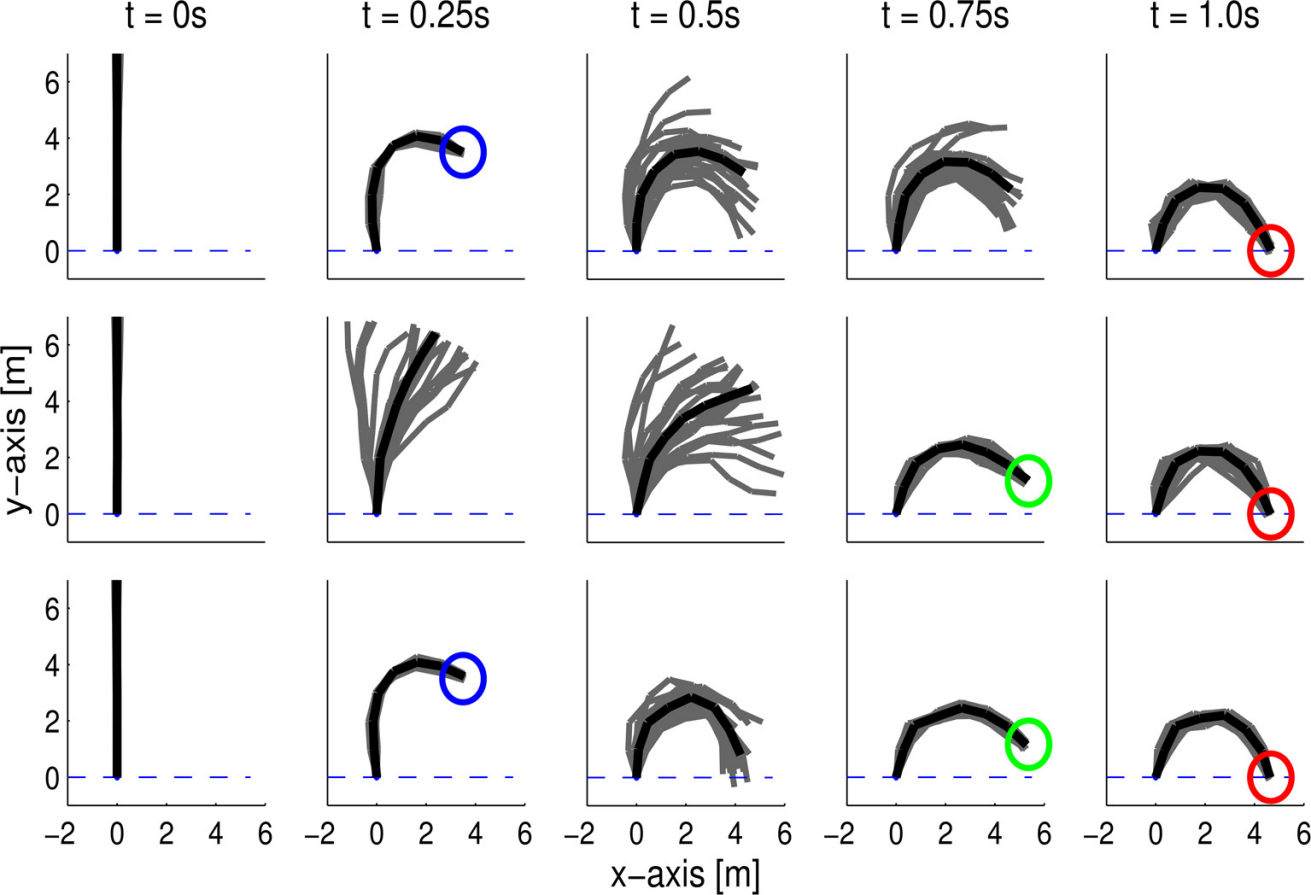
**A 7-link planar robot has to reach a target position at *T* = 1.0 s with its end-effector while passing a via-point at *t*_1_ = 0.25 s (top) or *t*_2_ = 0.75 s (middle)**. The plot depicts the mean posture of the robot at different time steps (black) and samples generated by the ProMP (gray). The demonstrations have been generated by an optimal control law. The ProMP approach was able to exactly reproduce the coupling of the joints from the demonstrations. The combination of both learned ProMPs is shown in the bottom. The resulting movement reached both via-points with high accuracy.

In this task, a seven link planar robot has to reach different target positions in end-effector space at the final time point *t*_*T*_ and at a via-point *t*_*v*_. We generated the demonstrations for learning the MPs with an optimal control law, (Toussaint, 2009) and adding noise to the control outputs. In the first set of demonstrations, the robot reached a via-point at *t*_1_ = 0.25 s with its end-effector. We used 10 normalized Gaussian basis functions per joint, resulting in a 70-dimensional weight vector. As we learned a single distribution over all joints of the robot, we can also model the correlations between the joints. These correlations are required to learn to reach a desired via-point in task space. The reproduced behavior with the ProMPs is illustrated in Figure 8 (top). The ProMP exactly reproduced the via-points in task space. Moreover, the ProMP exhibited the same variability in between the time points of the via-points. It also reproduced the coupling of the joints from the optimal control law, which can be seen by the small variance of the end-effector in comparison to the rather large variance of the single joints at the via-points. We also used a second set of demonstrations where the first via-point was located at time step *t*_2_ = 0.75, which is illustrated in Figure 8 (middle). We co-activated the ProMPs learned from both demonstrations. The robot could accurately reach both via-points at *t*_1_ = 0.25 and *t*_2_ = 0.75, see Figure 8 (bottom).

#### 3.3.2. Experimental evaluation of the combination of simultaneous objectives - robot hockey

**Figure 9:**
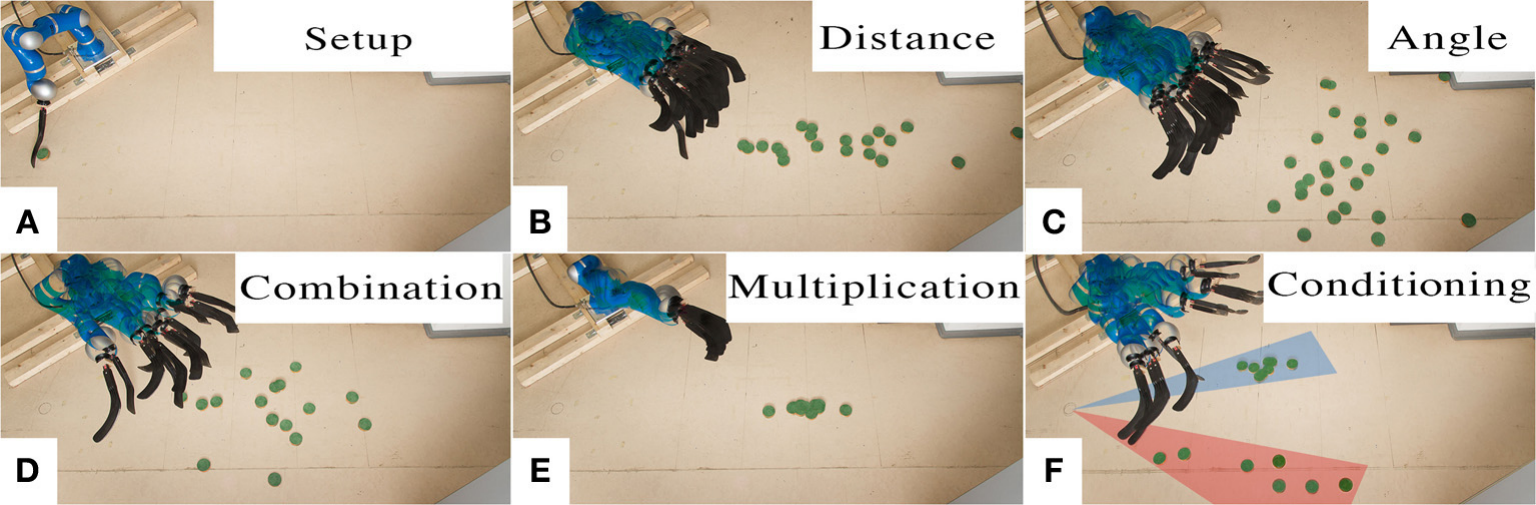
**Robot Hockey**. The robot shoots a hockey puck. The setup is shown in **(A)**. We demonstrate ten straight shots for varying distances and ten shots for varying angles. The pictures show samples from the ProMP model for straight shots **(B)** and angled shots **(C)**. Learning from combined data set yields a model that represents variance in both, distance and angle **(D)**. Multiplying the individual models leads to a model that only reproduces shots where both models had probability mass, in the center at medium distance **(E)**. The last picture shows the effect of conditioning on only left or right angles, the robot does not shoot in the center any more **(F)**.

In this task, the robot again has to shoot a hockey puck in different directions and distances. The task setup can be seen in Figure 9A. We record two different sets of demonstrations, one that contains straight shots with varying distances, while the second set contains shots with a varying shooting angle and almost constant distance. Both data sets contained ten demonstrations each. Sampling from the two models generated by the different data sets yields shots that exhibit the demonstrated variance in either angle or distance, as shown in Figures 9B,C. When combining the data sets of both primitives and learning a new primitive, we get a movement which exhibits variance in both dimensions, i.e., angle and distance, see Figure 9D. When the two individual primitives are combined by a product of MPs, the resulting model shoots only in the center at medium distance, i.e., the intersection of both MPs, see Figure 9F.

In this section, we present two experiments that we performed with the ProMP approach. As we focused on the representation of the individual building blocks, we evaluated the new representation without the use of reinforcement learning and learned the ProMPs by imitation. In our experiments, we illustrate how to use conditioning as well as co-activation of the building blocks.

## 4. Conclusion and future work

Using structured, modular control architectures is a promising concept to scale robot learning to more complex real-world tasks. In such a modular control architecture, elemental building blocks, such as movement primitives, need to be adapted, sequenced or co-activated simultaneously. In this paper, we presented a unified data-efficient policy search framework that exploits such control architectures for robot learning. Our policy search framework can learn to select, adapt and sequence parametrized building blocks such as movement primitives while coping with the main challenges of robot learning, i.e., high dimensional, continuous state and action spaces and the high costs of generating data. Moreover, we presented a new probabilistic representation of the individual building blocks which show several beneficial properties. Most importantly, they support efficient and principled ways of adapting a building block to the current situation and we can co-activate several of these building blocks.

Future work will concentrate on integrating the new ProMP approach into our policy search framework. Interestingly, the upper-level policy would in this case directly specify the trajectory distribution. The lower level control policy is automatically given by this trajectory distribution. We will explore to incorporate the co-activation of individual building blocks also in our policy search framework. Additional future work will concentrate on incorporating perceptual feedback into the building blocks and using more complex hierarchies in policy search.

### Conflict of interest statement

The authors declare that the research was conducted in the absence of any commercial or financial relationships that could be construed as a potential conflict of interest.

## Footnotes

^1^Alternatively, we can assume that we use inverse dynamics control on the robot, and, hence, the idealized dynamics of the robot are given by a linear system. Such an approach is, for example, followed by the DMPs that also assumes that the underlying dynamical system, that represents the robot, is linear.
